# Emergence of black spot syndrome in Caribbean reefs: a century of fish collections reveal long-term increases in *Scaphanocephalus* infection

**DOI:** 10.1098/rspb.2024.2065

**Published:** 2024-11-13

**Authors:** Pieter T. J. Johnson, Rémon J. Malawauw, Julia Piaskowy, Dana M. Calhoun, Zachary Kohl, Lars J. V. ter Horst, Derek A. Zelmer

**Affiliations:** ^1^ Ecology and Evolutionary Biology, University of Colorado, Boulder, CO, USA; ^2^ Department of Freshwater and Marine Ecology, Institute for Biodiversity and Ecosystem Dynamics, University of Amsterdam, Amsterdam, The Netherlands; ^3^ Department of Biology, Chemeketa Community College, Salem, OR, USA; ^4^ Department of Biological, Environmental and Earth Sciences, University of South Carolina Aiken, Aiken, SC, USA

**Keywords:** emerging disease, host-parasite interaction, biorepositories, historical parasitology, coral reefs, reef herbivores

## Abstract

Despite evidence that certain diseases of marine wildlife are increasing, long-term infection data are often lacking. Archived samples of hosts from natural history collections offer a powerful tool for evaluating temporal changes in parasitism. Using vouchered fish collections from the Southern Caribbean, we investigated long-term (1905–2022) shifts in infections by the trematode *Scaphanocephalus* spp., which causes black spot syndrome (BSS) in reef fishes. Examination of 190 museum-preserved fishes from Curaçao and Bonaire revealed that *Scaphanocephalus* infections are not new, with histologically confirmed detections from as early as 1948. However, *Scaphanocephalus* was rare among archival surgeonfish and parrotfishes, with an infection prevalence of <10% and an average abundance of 0.25 metacercariae per fish. Contemporary collections of 258 ocean surgeonfish and parrotfishes (7 species) supported a 7-fold higher prevalence (71%) and a 49-fold higher abundance (12.1). These findings offer evidence that infections by *Scaphanocephalus* spp. have increased substantially over the past century and underscore the value of biological repositories in the study of emerging parasites within marine ecosystems. We emphasize the need for additional research to evaluate the geographical extent of BSS emergence, test proposed hypotheses related to shifts in host density or environmental characteristics and assess the consequences for affected species.

## 1. Introduction

Emerging infections are among the most challenging and pervasive threats confronting marine ecosystems (e.g. [1]). Over the past four decades, an increasing number of pathogens and diseases have been reported to affect corals, echinoderms, molluscs, crustaceans, fishes, sea turtles and marine mammals (e.g. [2–4]). Some of these diseases have had profound ecological impacts, leading to shifts in biodiversity, species interactions or ecosystem structure [5]. Disease-driven losses of long-spined sea urchins (*Diadema antillarum*) from the Caribbean in the 1980s, for instance, contributed to increased algal growth and subsequent coral declines that persist to this day [6]. More recently, emergence of sea star wasting syndrome in temperate ecosystems has devastated important predators such as sunflower stars (*Pycnopodia helianthoides*), leading to increases in herbivory that can threaten kelp forest communities [7].

The cryptic nature and episodic dynamics of pathogens often impede both our understanding of host–parasite interactions and our ability to manage disease-related threats [8]. This is particularly true in ocean ecosystems, for which the geographic scope and biological complexity compound the surveillance problem [9]. For most taxa, particularly those not harvested commercially, we lack the long-term data necessary to evaluate whether an infection is emerging and, if so, at what rate or in which locations [10]. This knowledge gap, coupled with a lack of detailed information on shifts in environmental drivers, often challenges efforts to understand the mechanisms of disease emergence and develop effective interventions to mitigate their consequences. The fact that many marine pathogens use multiple hosts, either alternatively or sequentially in their life cycles, can further complicate efforts to identify emergence aetiologies as changes in any number of species or trophic levels may be contributing.

A powerful tool in the study of disease emergence is the use of archival museum collections from natural history museums [11]. Preserved hosts—and the parasites embedded within them—can function as ‘ecological time capsules’ to provide novel insights into changes in infection across space or through time [12]. In studies of human epidemiology, for instance, vouchered museum samples and biorepositories have helped understand infection origins or changes for diseases such as bubonic plague, Spanish flu, Lyme disease and hantavirus pulmonary syndrome [13–16]. Museum collections have yielded similarly valuable information about wildlife infections of fishes, amphibians, birds, zooplankton and molluscs [17–20]. Yet despite growing concerns about emerging infections in marine ecosystems, museum collections remain underutilized in the study of long-term changes in parasitological infections from marine habitats, and we are aware of no such examples involving coral reef ecosystems. The few examples from marine systems illustrate both the importance and utility of this approach. Using archived fish samples (1930–2016), for example, Howard *et al*. [21] reported long-term increases in nematode infection (*Clavinema mariae*) in the economically important English sole (*Parophrys vetulus*) from Puget Sound, USA.

**Figure 1.**
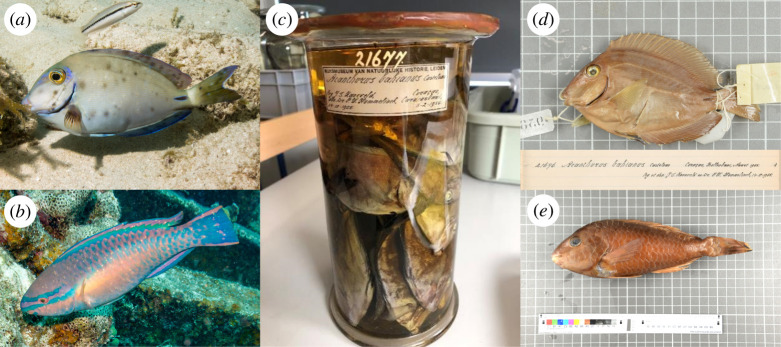
Examples of *Scaphanocephalus*-induced pigmented dermatopathies (black spot syndrome) in (*a*) ocean surgeonfish (*Acanthurus tractus*) and (*b*) princess parrotfish (*Scarus taeniopterus*). Encysted parasites and the surrounding lesions can appear black or white depending on the fish species and location. Infections in recently collected fish were compared with those from archival collections from the Naturalis Biodiversity Center (*c*) for both surgeonfish (*d*) and parrotfish (*e*). Image credits: (*a*) Randall Spangler, (*c*)–(*e*) Rémon Malawauw.

**Figure 2.**
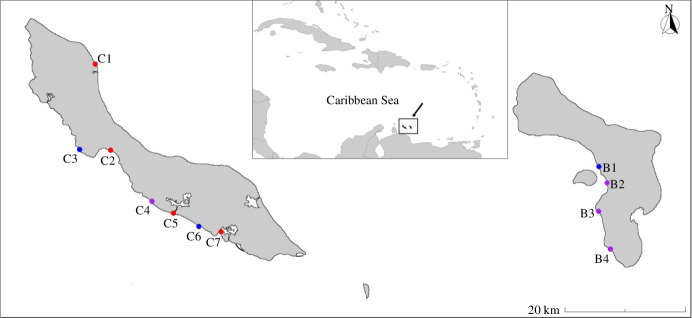
Curaçao and Bonaire are oceanic islands located in the southern Caribbean Sea. The locations of historical fish collections are depicted with red circles, the locations of contemporary collections in blue circles and locations with both historical and contemporary collections are depicted with a purple circle. Additional information for specific collection sites, including the corresponding codes, is presented in electronic supplementary material, table S2.

Here, we combine examinations of archived museum samples (1905–1966) with contemporary sampling to evaluate the emergence of a recently reported phenomenon affecting Caribbean reef fishes: black spot syndrome (BSS). Since 2011, increasing reports of dermal lesions and pigmented dermatopathies in the skin and fins of keystone herbivores, such as surgeonfishes and parrotfishes (figure 1), have generated concerns over the potential consequences for reef ecosystems. The aetiological agent of this condition was recently identified as infection by the trematode parasite, *Scaphanocephalus expansus*, which cycles among marine molluscs, fishes and osprey (*Pandion haliaetus*) [22]. However, efforts to rigorously test whether the infection is new in the region or emerging have been limited by a lack of historical data, particularly at time scales of more than a few years [23]. To overcome this challenge, we examined archival surgeonfish (family Acanthuridae) and parrotfishes (family Labridae) from a unique historical collection maintained by the Naturalis Biodiversity Center in Leiden, The Netherlands. We focused on the Southern Caribbean islands of Bonaire and Curaçao (figure 2), which have been consistently identified as hotspots for BSS [23–25]. By comparing archival samples to recently collected fishes representing the same or taxonomically and ecologically similar species and locations, we tested for changes in *Scaphanocephalus* spp. infection prevalence and infection load through time, between islands, and among fish taxa. Given the importance of large-bodied herbivorous fish in regulating algal growth around coral reefs [26], these results have important implications for understanding potential threats to keystone species in coral reef ecosystems.

### (a) Black spot syndrome in the Caribbean

Research on BSS in the Caribbean is relatively recent and dispersed across diverse source types, underscoring the importance of synthesizing this information and contextualizing motivations for the current study. The earliest report of BSS in the Caribbean traces to an August 2011 post by the Caribbean Research and Management of Biodiversity (CARMABI) research station [27] and a follow-up news story in 2012 [28], which reported a high prevalence of black and white spots in surgeonfish, parrotfishes, hamlets and grunts around Curaçao. The cause of the dermal lesions was not identified, but observers speculated that parasites could be responsible [24]. Subsequent investigations began to quantify the phenomenon. During surveys for invasive lionfish, Rooijen & Roothans [29] documented dermatopathies in *Acanthurus tractus* across 21 sites at prevalence values up to 64%. Bernal *et al.* [24] reported dermal lesions in 37 fish species in Curaçao, with the highest frequency in ocean surgeonfish (*A. tractus*; 19.7%), but few to no detections in Belize and Mexico, respectively. More recently, de Wit & Johnson [25] used video-based transects to quantify BSS severity (lesions per fish) in *A. tractus* across 35 sites along the leeward coast of Curaçao. Overall, 70% of fish exhibited lesions, and the severity of lesions increased with nutrient run-off and fishing pressure while decreasing with wave intensity.

Observations from other Caribbean locations began to emerge shortly thereafter. At the Council on International Educational Exchange field station in Bonaire, student projects reported a high prevalence of BSS (approx. 80%) in *A. tractus* from the shallow waters near Kralendijk [30,31]. A 2015 study commissioned by the Dutch Government surveyed 16 040 fish and identified dermal lesions in 36 of 41 species, with some of the highest prevalences in *A. tractus* (57%) and *Sparisoma aurofrenatum* (46%) [32]. The severity of BSS in Bonaire decreased with depth and increased between surveys conducted in 2012 and 2017, respectively [23]. The frequency of affected fish was consistently greatest on the western coast near the urban center of Kralendijk, suggesting a potential link to water quality [23,32]. Beyond the Southern Caribbean, BSS has recently been reported in acanthurid fishes from Belize (approx. 60% [33]), The Bahamas [33], St Kitts (40–50% [34]), Saba and St Eustatius (0–28% [35]), Turks and Caicos [36] and the Windward Islands of the Lesser Antilles (2–13.4%; K. C. Kingon & M. Olton 2018, unpublished data). Using a search of web-archived images of *A. tractus*, Elmer *et al*. [23] identified evidence of BSS at 14 of 26 locations across the Caribbean dating back to 1985, for which the Southern Caribbean exhibited the highest prevalence values (78%).

Using genetics and morphological approaches, Kohl *et al*. [22] identified the black spots as the encysted larval form (metacercaria) of the opisthorchid trematode, *S. expansus*. Each pigmented dermatopathy, which can manifest as a black or white spot depending on the fish species and location within the host (figure 1), was associated with one or more metacercariae. The number of lesions observed *in situ* correlated positively with the number of encysted *Scaphanocephalus* spp., highlighting the utility of non-invasive approaches to assess infection based on external spot counts [22,37]. Parasites in this genus, which exhibit distinctive anterior ‘wings’, have complex life cycles involving a molluscan first intermediate host (as of yet unidentified for *Scaphanocephalus*), reef fishes as second intermediate hosts and predatory birds (primarily osprey, *P. haliaetus*) as definitive hosts [22]. The black spots on affected fish are hypothesized to increase their conspicuousness to osprey thereby enhancing the likelihood of transmission [22]. Prior records of this parasite in the region are rare; *Scaphanocephalus* spp. metacercariae have occasionally been reported at low prevalence from fishes along the Gulf of Mexico and parts of Florida [38–40], but we are aware of only a single previous record from the Caribbean [41]. In contrast, contemporary evidence indicates that *Scaphanocephalus* spp. occur in a broad range of fish species that vary in ecology, life history and phylogeny [42–44]. In a survey of the reef fish community around Curaçao, *Scaphanocephalus* spp. metacercariae were detected in 29 of 41 species at loads of up to 564 cysts (based on dissections from one side of each fish [37]). Large-bodied species of lower trophic levels, such as parrotfishes and surgeonfishes, were among the most infected, with no significant influence of phylogeny.

Taken together, these observations indicate that infections by *Scaphanocephalus* spp. trematodes and the resulting cutaneous lesions occur widely among tropical and subtropical regions yet vary substantially across fish species and locations. Although multiple types of infections have the potential to cause black spots on the fins and scales of marine fishes, including protozoans, turbellarians (e.g. *Paravortex* spp.) and other digenetic trematodes (e.g. *Cryptocotyle lingua*, *Liliatrema skrjabini*), the types of lesions *Scaphanocephalus* induces tend to be distinctive in appearance and location. Within the Southern Caribbean (Curaçao and Bonaire), thus far only *Scaphanocephalus* spp. infections have been found in association with hyperpigmented dermatopathies (examination of 504 fish from 42 species [37]). While initially identified as *S. expansus*, recent genetic analyses have revealed the presence of multiple species, even within the Caribbean [34,45,46], and we therefore use ‘*Scaphanocephalus* spp.’ pending additional taxonomic revisions. It is further apparent that observations of BSS and research on *Scaphanocephalus* spp. has increased considerably over the past decade. However, rigorous data to assess long-term changes in infection have been lacking, particularly at temporal scales beyond a few years. Here we address this gap by combining examinations of archival fish collections with contemporary sampling to quantify changes in *Scaphanocephalus* spp. infection over the past century.

## 2. Material and methods

### (a) Study system

Curaçao and Bonaire are small oceanic islands in the Leeward Antilles (444 and 288 km^2^, respectively) of the southern Caribbean Sea (figure 2). Each island is rimmed by a fringing coral reef with distinct leeward and windward coastlines. The leeward reef habitat is characterized by shallow sand flats extending approximately 30–200 m before reaching the coral reef crest and subsequent slope. The reefs of both islands represent biodiversity hotspots and support some of the highest coral cover in the Caribbean Sea, despite widespread regional declines in coral reefs [47]. They also support robust populations of parrotfishes (family Labridae) and surgeonfishes (family Acanthuridae) within the shallow reef habitats surrounding each island [48]. By limiting the growth and establishment of turf- and macroalgae, these large-bodied herbivores help to maintain conditions conducive for coral recruitment and thereby enhance reef resilience to additional stressors [26,49]. The removal of carbonate materials from the reef (i.e. bioerosion) by excavating parrotfish also plays an important role in shaping coral growth, species composition, nutrient cycling and sedimentation rates [49]. Overfishing of such herbivores in the Caribbean has been implicated as a major contributing factor in phase shifts from coral- to algal-dominated stages, which are notoriously difficult to reverse [50].

Curaçao and Bonaire were also the focus of extensive historical collections associated with characterizing organismal diversity and natural history, including samples of shallow-water reef fishes, creating a valuable opportunity to assess long-term changes in parasite infection. We focus here on collections by J. Boeke (1904–1905, Zoölogisch Museum Amsterdam), C. J. van der Horst (1920, University of Amsterdam), Jacques S. Zaneveld (1955, Caribbean Marine Biological Institute), Pieter Wagenaar Hummelinck (1948–1955, University of Utrecht) and Jan H. Stock (1958, University of Amsterdam; [51–55]). Samples from these collections are stored either individually or in batches as ‘wet specimens’ (i.e. fluid preserved), which functionally preserves fish hosts as well as the parasites encapsulated within them (e.g. [12]). Additional information on these collections is provided in the electronic supplementary material.

### (b) Examination of archived fish from Curaçao and Bonaire

To obtain historical information on the presence and abundance of *Scaphanocephalus* spp. infections, we examined fish collections housed at the Naturalis Biodiversity Center in Leiden, The Netherlands (figure 1). Naturalis is one of the largest natural history museums and maintains extensive collections of marine fishes from the Dutch Caribbean, including Curaçao and Bonaire. We examined fish species that supported infection in contemporary collections, including samples of ocean surgeonfish (*Acanthurus tractus*) and parrotfishes (family Labridae): striped parrotfish (*Scarus iseri*), princess parrotfish (*Scarus taeniopterus*), redband parrotfish (*Sparisoma aurofrenatum*), redtail parrotfish (*Sparisoma chrysopterum*) and stoplight parrotfish (*Sparisoma viride*). Very small fish (<4.5 cm in total length) were excluded. We updated historical species identification to follow contemporary taxonomy (World Register of Marine Species [56]). The sample sizes, collection dates, average sizes, locations and accession numbers for each collection are listed in electronic supplementary material, tables S1 and S2, and summarized in table 1.

**Table 1.** Summary information on surgeonfish and parrotfish species sampled from Bonaire and Curaçao. Observations are divided into historical (1905–1966) and contemporary (2017–2022) specimens. For each species and time period, the sample size, prevalence of *Scaphanocephalus* spp. infection, and average abundance of metacercariae per host (infection load) are presented. Note that, in statistical analyses, fish species identity was nested within family (Acanthuridae and Labridae) and treated as a random intercept term owing to variable sample sizes and survey dates. See electronic supplementary material, table S1 for more detailed information.

species	historical (1905–**1966**)			contemporary (2017–**2022**)		
Curaçao	*n*	prev. (%)	abun.	*n*	prev. (%)	abun.
*A. tractus*	87	3.0	0.06	65	72	8.77
*Sp. aurofrenatum*	6	0.0	0	53	85	31.77
*Sp. chrysopoterum*	56	4.0	0.04	22	73	4.32
*Sp. rubripinne*	—	—	—	2	50	1
*Sp. viride*	—	—	—	19	26	3.21
*Sc. iseri*	9	0.0	0	17	53	3.29
*Sc. taeniopterus*	1	0.0	0	45	71	4.87
Bonaire						
*A. tractus*	12	50	1.83	31	77	7.26
*Sp. aurofrenatum*	—	—	—	3	100	64.67
*Sp. chrysopterum*	9	78	1.78	1	100	4
*Sp. radians*	1	0.0	0	—	—	—
*Sp. viride*	1	0.0	0	—	—	—
*Sc. taeniopterus*	1	0.0	0	—	—	—

Each fish was photographed prior to dissection, from which total length was measured using ImageJ [57]. Because *Scaphanocephalus* spp. metacercariae occur predominantly in the fins and surficial epidermis of infected fish, we first examined the entire external surface of the fish on both sides for encysted parasites before inspecting the pectoral, caudal and dorsal fin rays using a combination of transmitted and reflected illumination. For a subset of archival surgeonfish (*n* = 21) from Curaçao, we removed a 3 cm × 3 cm sample of the skin and underlying tissues from one side of the body to quantify encysted metacercariae. Although this tissue window was smaller than those used for recently collected fish (see below), it was selected to limit damage to historical samples while still offering an opportunity to detect subsurface infections not visible from external examination. Isolated metacercariae were stored initially in 95% ethanol, post-fixed in Bouin’s fluid, dehydrated through an ethanol series, and cleared and mounted in cedar oil for observation (Hoffman modulation contrast microscopy) and measurement.

### (c) Examination of contemporary fishes from Curaçao and Bonaire

To detect and quantify *Scaphanocephalus* spp. among contemporary fish, we collected ocean surgeonfish (*A. tractus*) and parrotfishes (*Sc. iseri*, *Sc. taeniopterus*, *Sp. aurofrenatum*, *Sp. chrysopterum* and *Sp. viride*) from shallow depths (1–10 m) at four sites around Bonaire and three sites around Curaçao. Collections were made in 2017 (Bonaire) and in 2022 (Curaçao) using a broad range of capture methods (see [22,37] for additional details). After collection, we examined the upper fins (dorsal, pectoral and caudal fins) and skin on the upper half of the body. On a randomly selected side of each fish (left or right), a window of skin (and the underlying tissue) extending from immediately posterior of the operculum to the base of the caudal fin and dorsally from the body midline to the base of the dorsal fin was removed. Under a stereomicroscope, the skin and fins were examined using transmitted light, whereas underlying muscle was inspected for metacercariae using reflected light. The surface area of the dissection window and the total length of the fish were measured from images using ImageJ. A haphazard subset of metacercariae from each collection was mechanically excysted to confirm the presence of the anterior wings that are morphologically diagnostic for the genus *Scaphanocephalus* [22]. Among recently captured fish, metacercariae of *Scaphanocephalus* spp. were also easily differentiated from those of other trematodes based upon the presence of a thick, discoidal, fibrous capsule surrounding the parasite cyst, an obvious gap between the fibrous capsule and the parasite cyst, and extensive folding of the metacercaria body that obscures the anatomy.

### (d) Comparison of parasite morphometrics between recent and historical samples

While fresh metacercariae can be excysted and easily verified as *Scaphanocephalus* spp., this was not always possible for historical specimens, some of which were >50 years old and brittle. Long-term storage or fixation in formalin can further challenge efforts to extract useable genetic material. We, therefore, used two morphological approaches to validate the identity of historical metacercariae as *Scaphanocephalus* spp. First, we measured key morphological traits of the parasite and cyst, including maximum inside diameter of the fibrous capsule, largest diameter perpendicular to that maximum, maximum and minimum thicknesses of the capsule wall, length of the parasite cyst within the capsule, greatest cyst width perpendicular to the length and thickness of the wall enclosing the parasite cyst. All measurements were made from digital images using GIMP [58]. Cysts that were clearly identifiable as species other than *Scaphanocephalus* spp. were stained in acetocarmine and mounted in Damar for additional visualization.

Second, we used serial sectioning to examine the internal structures of encysted metacercariae. Specimens in cedar oil were secondarily cleared in toluene, stained with eosin toluol to improve visibility, embedded in paraffin, sectioned at 5–7 μm, stained with Lillie–Meyer haematoxylin and counterstained with eosin xylol (McLean 1934, as cited in [59]). We examined sections for seven marker characteristics established from whole mounts of known *Scaphanocephalus* spp. metacercariae: (i) a fibrous, eosinophilic capsule surrounding the cyst, (ii) a moderately basophilic cyst wall, (iii) extensive folding of the metacercaria, (iv) exceptionally thin (1 cell thick) cross-section at anterior of the metacercaria, (v) an oral sucker associated with the thin cross-section of the body, (vi) an acetabulum associated with the thick cross-section of the body and (vii) flanked by visibly sinuous ceca. The allometric relationships of any cysts that could not be sectioned (due to calcification, gas bubbles or tissue deterioration) were compared with those of sectioned cysts identified as *Scaphanocephalus* spp. using linear modelling. Cyst wall thickness was compared between the two groups of cysts using a two-sample *t*‐test.

### (e) Statistical analysis

We used a generalized linear mixed modelling (GLMM) framework to test for changes in infection prevalence and infection load between recent and historical fish samples because these models can incorporate fixed and random effects, handle a range of error distributions and are robust to unbalanced sampling designs [60,61]. Prevalence (the presence or absence of *Scaphanocephalus* spp. metacercariae in a fish) was modelled as a Bernoulli distribution with a logit-link function, while infection load (the number of metacercariae in a given host) was modelled using a negative binomial distribution (‘nbinom2’ in glmmTMB). Models included island (Curaçao or Bonaire), fish total length, family (Acanthuridae or Labridae) and sample year. We incorporated random intercept terms for fish species (*n* = 8) and collection locations (*n* = 13). Numeric terms were centred and scaled prior to inclusion. Because sampling through time was uneven both in frequency and taxonomic coverage, we conducted a second set of models in which time was treated as a dichotomous variable (historical [1905–1966] versus contemporary [2017–2022]). Models were implemented using the glmmTMB package in R [62] and were assessed for fit to the data (marginal and conditional *R*^2^), collinearity, zero inflation, outliers and overdispersion. Spatial autocorrelation in the residuals was examined using Moran’s *I* and temporal autocorrelation was tested using a Durbin–Watson test (for additional details on model construction and diagnostics, see electronic supplementary material).

Because of slight differences in the protocols used to examine contemporary versus archived fishes, we restricted the statistical analysis to only consider *Scaphanocephalus* spp. metacercariae detected in the fins on one side of the fish, including the dorsal fin, the caudal fin and one of the pectoral fins (left or right). Museum fish in which the dorsal fin could not be extended (*n* = 7) were omitted from the analysis. For the subset of museum fish in which we examined a window of skin and muscle, we conducted an additional analysis comparing the abundance of parasites within the window between recent (*n* = 47) and historical fish (*n* = 22). An offset term was included for the area of the window to account for variation in tissue examined. The fixed and random effects were identical to those presented above, with the exception that this analysis focused only on *A. tractus* from Curaçao.

## 3. Results

**Figure 3.**
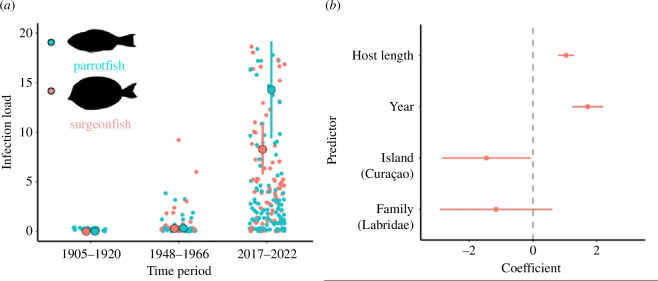
(*a*) Both the presence and abundance of *Scaphanocephalus* spp. metacercariae increased substantially between historical collections (1905–1920, 1948–1966) and contemporary samples (2017–2022) of reef fish. Specimens of ocean surgeonfish (*Acanthurus tractus*) are depicted in peach, while parrotfishes (*Scarus* and *Sparisoma* spp.) infections are shown in teal. The earliest confirmed infections of *Scaphanocephalus* spp. were detected in 1948. Error bars represent 95% confidence intervals around the mean. (*b*) Coefficient plot illustrating the effects of predictor terms on the average number of *Scaphanocephalus* metacercariae per fish host from a GLMM with size of the host, year of collection, island (Curaçao versus Bonaire) and family (Acanthuridae versus Labridae). Error bars represent 95% confidence intervals. Numeric predictor terms were centred and scaled prior to inclusion. The vertical dashed line at zero indicates a lack of any effect. Coefficient values presented are from a model in which year was treated as a continuous predictor, although results are nearly identical when time was treated as dichotomous (historical [1905–1966] and contemporary [2017–2022]), see electronic supplementary material.

Among historical fish collections, infection by *Scaphanocephalus* spp. was both infrequent and, when present, of low intensity relative to contemporary collections. In total, we examined 190 historically collected surgeonfish and parrotfishes captured in 1905 (*n* = 18), 1920 (*n* = 17), 1948 (*n* = 21), 1955 (*n* = 124), 1958 (*n* = 7) and 1966 (*n* = 3; see electronic supplementary material, table S1). This included 166 fish from Curaçao and 24 from Bonaire, of which 56.3% were surgeonfish and 43.7% were parrotfishes. For contemporary collections, we quantified infections among 35 fish from Bonaire and 223 from Curaçao. Collection year had a strong, positive effect on both the prevalence and abundance of *Scaphanocephalus* spp. infection (prevalence: binomial GLMM: scale [year] = 1.934 ± 0.349, *z* = 5.534, *p* < 0.000001, *R*^2c^ = 0.75; parasite abundance: negative binomial GLMM: scale [year] = 1.728 ± 0.247, *z* = 6.988, *p* < 0.000001, *R*^2c^ = 0.81; *n* = 437; figure 3*a* electronic supplementary material, table S3). Overall, 9.8% of historical fish (Wilson score 95% CI [6.3%−14.9%]) were infected with *Scaphanocephalus* spp., with an average abundance of 0.27 for surgeonfish (range: 1–9) and 0.21 for parrotfishes (range: 1–4). The earliest detected infections were from 1948, involving *A. tractus* and *Sp. chrysopterum* from Bonaire. Fish from Bonaire supported significantly higher average parasite abundance relative to those from Curaçao (*p* = 0.036). There were no significant main effects of fish family (Labridae versus Acanthuridae; figure 3*b*). Model diagnostics indicated no significant evidence of overdispersion, collinearity (all VIFs <2), zero-inflation, outliers, spatial or temporal autocorrelation (all *p* > 0.05). Treating time as a dichotomous variable (historical versus contemporary) yielded nearly identical results (see electronic supplementary material, table S4 and electronic supplementary material for full model results).

Among recently collected fish (2017–2022), 74% of surgeonfish (Wilson score 95% CI [64.4%−81.7%]) and 69% of parrotfishes (Wilson score 95% CI [61.6%−75.7%]) were infected, with average abundance ± 1 s.e. values of 8.28 ± 1.28 (range: 1−68) and 14.29 ± 2.45 (range: 1−180), respectively (*n* = 258; all infection values for archival and contemporary fish were tallied from one side of the host only, not including any subsurface dissections). This represented a 7-fold increase in infection prevalence and a 49-fold increase in average infection load between historical and recent collections (table 1). Such a difference is noteworthy given that historical samples included fishes from some of the same locations as recent observations (e.g. Piscaderabaai in Curaçao, Kralendijk and The Lake in Bonaire; figure 2). Fish length also positively predicted infection prevalence (scale[Length] = 1.238 ± 0.225, *z* = 5.510, *p* < 0.000001) and abundance (scale[Length] = 1.040 ± 0.131, *z* = 7.957, *p* < 0.000001; figure 3*b*). Other large metacercariae, such as those of *Stephanostomum* spp., were relatively rare and encountered in <2% of surveyed fish.

For the subset of historical surgeonfish in which a window of muscle and skin was dissected to quantify infection, a comparable increase in parasite abundance was observed through time (negative binomial GLMM: scale[Year] = 2.095 ± 0.350, *z* = 5.998, *p* < 0.000001; *n* = 68). Only a single metacercaria was observed deep into the dermis in historical specimens in which a tissue window was examined (average abundance = 0.04); however, 37 of the 47 fish from 2022 were infected with one or more *Scaphanocephalus* spp. in skin, subcutaneous tissue or muscle (average abundance = 14.8; range 1–128). In only two instances were conspicuous black spots detected in historical fish prior to microscope-based examinations, both of which involved *Sp. chrysopterum* and were linked to *Scaphanocephalus* infection. Fish total length did not significantly influence parasite abundance with the subset of individuals with a tissue window dissected (*p* > 0.1).

**Figure 4.**
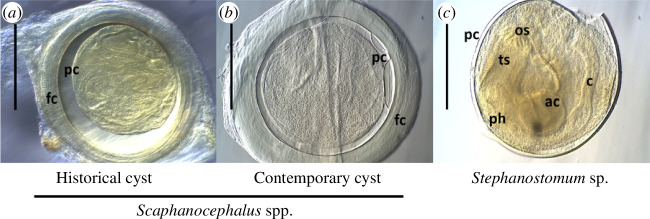
Hoffman modulation contrast images of cedar oil-cleared parasite cysts recovered from fish specimens. Scale bars are 500 μm. (*a*) *Scaphanocephalus* spp. metacercaria with parasite cyst wall (pc) enclosed within a fibrous capsule (fc). (*b*) *Scaphanocephalus* spp. metacercaria from a recently collected fish for comparison. (*c*) *Stephanostomum* spp. metacercaria within the parasite cyst (pc), showing two rows of circumoral spines surrounding the oral sucker (os), tegumental spines (ts) and an elongated pharynx (ph). The acetabulum (ac) and cecum (c) are also visible.

**Figure 5.**
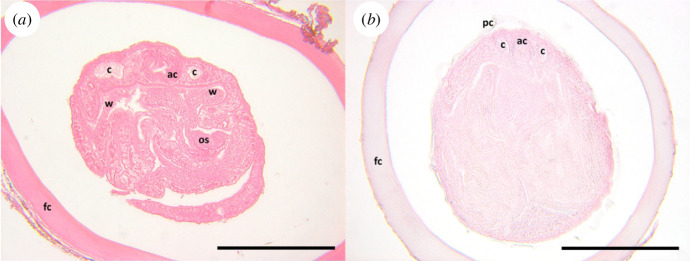
Haematoxylin and eosin-stained sections through *Scaphanocephalus* spp. cysts from museum specimens showing the characteristics used for taxonomic diagnosis. Scale bars are 200 μm. (*a*) Section showing acetabulum (ac), ceca (c), fibrous capsule (fc), oral sucker (os) and thin ‘wing’ (w). Lack of nuclear staining is the result of tissue degradation. (*b*) Section showing acetabulum (ac), ceca (c), fibrous capsule (fc) and parasite cyst wall (pc).

Of the 54 metacercariae isolated from archival fish hosts, 45 were positively identified as *Scaphanocephalus* spp. based on serial sectioning (*n* = 27) or allometric analysis (*n* = 18). The remaining nine were metacercariae of *Stephanostomum* spp., as determined by the presence of tegument spines, two alternating, uninterrupted rows of spines on the terminal oral sucker and an elongated prepharynx and pharynx (figure 4). None of the *Stephanostomum* cysts was enclosed within a fibrous capsule (as is typical for *Scaphanocephalus*; figure 4). Variation in fixation, preservation and condition of the specimens prior to preservation influenced section quality, and, as a result, the number of outlined *Scaphanocephalus* markers that could be observed in sectioned metacercariae (figure 5). All seven characters were visible in 12 of the 27 sectioned metacercariae identified as *Scaphanocephalus*, and four or more characters were visible in an additional 11 specimens (electronic supplementary material, figure S1). The four remaining specimens identified based on two (*n* = 2) or three (*n* = 2) characters were all isolated from fish that had additional metacercariae sectioned and identified as *Scaphanocephalus* spp.

The remaining 18 museum-collected metacercariae were not sectioned due to calcification or gas bubbles within the capsule. Analysis of their allometric relationships provided evidence they were also *Scaphanocephalus*, and all were collected from fish infected with other metacercariae identified as *Scaphanocephalus* by serial sectioning. Comparison of the allometry for unsectioned cysts with those specimens confirmed as *Scaphanocephalus* spp. by sectioning showed close agreement between: the inside diameters of the fibrous capsule (electronic supplementary material, figure S2A), the thickness measurements of the fibrous capsule (electronic supplementary material, figure S2B), the lengths and widths of the parasite cyst (electronic supplementary material, figure S2C), the maximum inside diameter of the fibrous capsule and the width of the parasite cyst (electronic supplementary material, figure S2D). For all relationships, linear modelling showed no significant effect of cyst treatment (sectioned versus not sectioned; *t* ranging from 0.023 to 0.802; *p* ranging from 0.427 to 0.982), or its interaction with allometry (*t* ranging from 0.125 to 1.112; *p* ranging from 0.273 to 0.901). There was also no significant difference in mean cyst wall thickness between sectioned and unsectioned metacercariae (*t* = 0.209; *p* = 0.836; electronic supplementary material, figure S1B).

## 4. Discussion

Our results provide quantitative evidence that infections by the trematode parasite *Scaphanocephalus* spp. are emerging among reef fishes in the Southern Caribbean. By examining 441 individual fish of seven species, we found that *Scaphanocephalus* spp. metacercariae were rare in archival fish collections (1905–1966) and, when present, of low intensity, such that contemporary samples (2017–2022) supported a 7-fold higher prevalence and a 49-fold higher average infection load. Across all sites, species and years, only 9.8% of archival fish showed signs of *Scaphanocephalus* spp. metacercariae, relative to 71% of recently collected fish. The magnitude of increase in infection was broadly consistent between islands and fish families (Acanthuridae and Labridae), although the sample sizes for Bonaire were lower than for Curaçao. Historical samples included a range of time periods, collectors, species and locations, helping to reduce the likelihood that observed differences stemmed from idiosyncrasies of a particular collection event or collector. In several cases, contemporary fish were collected from the same sites as historical surveys (e.g. Piscaderabaai in 1958, 1966 and 2022, Kralendijk in 1948 and 2017, and The Lake in 1905 and 2017).

This study yielded the earliest known records of *Scaphanocephalus* from fish hosts in the Caribbean and affirmed that the infection is not new to the region, even if it has increased significantly in recent decades. While none of the 34 fish collected from either island in 1905 or 1920 were infected with *Scaphanocephalus* spp., infections were evident in multiple fish species (*A. tractus* and *Sp. chrysopterum*) from Bonaire in 1948. The earliest recorded infections in Curaçao were from 1955, for which 4 of 117 fish were infected, each with a single *Scaphanocephalus* metacercaria. Analysis of allometric relationships and serial sectioning of historically encysted metacercariae—including distinctive morphological traits of the parasite—confirmed their identity as *Scaphanocephalus* spp. (figures 4 and 5), highlighting the approach’s utility in analysing archival parasite samples. These findings parallel those of other emerging human and wildlife diseases, for which analyses of samples from biorepositories have revealed that the pathogens existed regionally prior to their observed emergence, including examples such as *Borrelia burgdorferi* and the amphibian chytrid fungus [20]. Without DNA analysis, however, it is unclear whether all samples represent the same species relative to recent collections. At least two species of *Scaphanocephalus* are known to occur within Caribbean fishes [34,45,46], and this number may increase with additional sampling. Thus, even while our results show the parasite has occurred in the region for at least 70 years, it is possible that changes in species composition or genotypes may underlie the observed emergence.

An important consideration when working with archival collections is the identification of sources of bias associated with the long-term storage of specimens [63]. If parasites become more challenging to detect or fail to preserve in long-stored tissues, what appear as long-term shifts in infection might be artifactual. Several lines of evidence suggest that our ability to detect *Scaphanocephalus* is robust, even in century-old samples. Trematodes generally, and those of *Scaphanocephalus* spp. in particular, have cysts that are thick-walled and highly durable, able to persist until strong acids within the gut of their definitive hosts release the encysted parasites. Their positioning under the host skin or within the fin rays also affords them protection from being brushed off during collection, handling or storage [12]. Fiorenza *et al*. [64] found that the protocols used by museums to preserve fish (formalin fixation and alcohol preservation) did not adversely affect parasite detection for 25 of 27 host–parasite pairs, including those involving trematode metacercariae. There is also little evidence that historical collectors sought fish from different habitats relative to recent specimens. While it is possible that some museum collectors intentionally avoided diseased fish, particularly if seeking archetypal vouchers, this is less likely given the diverse set of collectors, time periods and fish species included here.

We openly acknowledge, however, that variation in sampling methods or the specific habitats surveyed (including depth) could have contributed to the observed changes in *Scaphanocephalus* spp. infection through time. An examination of the main collectors from the archived samples [51–55] (electronic supplementary material) indicated that animals were generally batch-collected during expeditions to characterize local biodiversity and species’ distributions, obtained using non-selective capture methods (especially nets and traps) across different times of year, and sampled from shallow, nearshore habitats. Although this generally aligned well with sampling from the current study, which captured fish from depths of 1 to 10 m in nearshore waters, one important difference is that fish traps were not used in our contemporary collections. Nonetheless, we suggest that such differences and any information shortages are unlikely to be the primary drivers behind the observed infection increase given that: (i) the analysis included a range of historical locations and collectors over a 60 year period (1905–1966), (ii) the removal of specific collections and re-analysis of the data did not appreciably alter our results and (iii) the magnitude of the observed increase (7-fold increase in prevalence and 49-fold increase in abundance) represents a foundational shift beyond anything detected in archival collections. Moreover, recent studies using diver- and image-based surveys in Curaçao and Bonaire indicate that BSS is both highly prevalent (often >50%) and often severe (numerous parasite-induced lesions per fish; e.g. [22–25]), providing further evidence that the current, widespread prevalence of *Scaphanocephalus* contrasts markedly from historical samples.

Conspicuous spots associated with *Scaphanocephalus* infections have also recently been observed from tropical and subtropical fishes outside the Caribbean, raising questions about the geographic extent of emergence and its potential implications. In Saudi Arabia, approximately 23% of *Siganus argenteus* from fish markets were infected with *Scaphanocephalus* spp. and exhibited black spots over much of the body surface, similar to descriptions of BSS in the Caribbean [65]. A large-scale survey of reef fishes around the Great Barrier Reef in Australia reported multiple putative species of *Scaphanocephalus* within 20 fish species [44]. In 2015, fishermen around the Balearic Islands of the Mediterranean reported spots on the skin of pearly razorfish (*Xyrichtys novacula*) that were subsequently identified as *Scaphanocephalus* spp. Heavily infected fish exhibited lower body condition, increased oxidative stress reactions and higher immunological activity [66,67]. In Japan, where *Scaphanocephalus* infections in tropical fish have a longer history of research [42], inspections of parrotfishes from fish markets revealed that *Scaphanocephalus* infection prevalence varied from <1% to 38.5% among species [43,68]. Infection was associated with a significant reduction in the value of fish [69], illustrating the economic repercussions associated with infection.

One of the most pressing questions arising from these results is why *Scaphanocephalus* spp. infections have increased over the past century in the Southern Caribbean. We advance three, non-mutually exclusive possibilities. First, changes in resource or habitat availability may have promoted the first intermediate hosts’ population growth, creating a greater pool from which infective cercariae emerge. The specific identity of this host remains unknown, but comparisons with life cycles from other opisthorchioid trematodes suggest it is likely a snail in one of the following superfamilies: Cerithioidea, Truncatelloidea or Littorinoidea [70]. One of the most profound changes to Caribbean reefs over the past five decades has been the shift from coral- towards algal-dominated substrates [47], for which the large-scale deterioration of acroporid corals, losses of key herbivores such as *Diadema antillarum* and nutrient run-off into coastal waters around Curaçao and Bonaire have all contributed to an increase in fleshy algae on local reefs [71]. The increase of algal resources can promote the density of grazing aquatic snail hosts, their production of trematode infective stages and the likelihood of interaction with susceptible herbivorous fishes [72]. Among reef sites in Curaçao, the severity of BSS in *A. tractus* correlates positively with increasing nitrogen loads [25]. Many of these changes have occurred or intensified since the 1970s and are thus consistent with the timeline for *Scaphanocephalus* emergence, although uncertainty about the identity of the parasite’s first intermediate host currently limits a more in-depth examination of these hypotheses.

Second, observed increases in *Scaphanocephalus* spp. infections may stem from changes in the abundance or activity of osprey, including both the American (*P. haliaetus carolinensis*) and Caribbean (*P. halieatus ridgwayi*) subspecies. While many trematodes that mature in birds can infect a range of species, *Scaphanocephalus* spp. occurs almost exclusively in osprey [22]; thus, the abundance, infection prevalence and geographic distribution of osprey are important determinants of infections in fish. In North America, osprey numbers declined precipitously in the 1950s and 1960s due to the use of dichlorodiphenyl-trichloroethane (DDT) [73], which caused eggshell thinning, mortality and decreased reproductive success [74]. Osprey numbers have increased substantially since the banning of DDT in 1972, increased federal protection from the Migratory Bird Act and active construction and preservation of breeding habitats (e.g. nest platforms; [75]). Between 1981 and 2001, for example, the number of breeding osprey pairs in annual USA breeding bird surveys doubled from 8 000 to 16 000 [74], likely facilitating concurrent increases in *Scaphanocephalus* dispersal and population size. However, this would not explain why infections among archival fishes were rare in the first half of the twentieth century before DDT use was widespread, which is a period for which we know little about osprey population dynamics or their infection with *Scaphanocephalus* [74].

Finally, long-term warming of sea surface temperatures may promote infections through physiological or ecological mechanisms. In the Caribbean, regional warming began around 1915 and accelerated considerably in the 1980s [76]. This pattern has been particularly pronounced in the Southern Caribbean, where waters have warmed by 0.26°C per decade since 1981 [76]. The effects of warming on trematode infections are often multifaceted and can be challenging to predict. Experimental studies have shown that realistic increases in temperature can substantially enhance production and release of trematode infective stages (e.g. cercariae) while also extending the seasonal duration during which such stages are produced [77–79]. At the same time, however, warming can increase mortality of infected first intermediate hosts and reduce the lifespan of cercariae, emphasizing the importance of experimental studies to assess the net influence of temperature on *Scaphanocephalus* transmission. Such effects can often be nonlinear and dependent on the taxon and degree of temperature change [77]. For instance, using museum-preserved fish samples from Puget Sound, USA, over a 130 year period, Wood *et al.* [80] found that progressive warming correlated with decreased infections of many digenetic trematodes, whereas parasites with more direct life cycles (one or two obligate hosts) exhibited few directional changes.

Ongoing observations of emerging infections in marine ecosystems have galvanized efforts to understand both the drivers and consequences of disease for wildlife conservation, ecosystem function and economic production. A key prerequisite to such investigations is determining whether the infectious agent is new to the region and quantifying how infection levels have changed through time, among species and across locations. Natural history collections offer an important tool for addressing such questions, for which the current study of *Scaphanocephalus* spp. was particularly well suited because of (i) the availability of an extensive historical collection across locations, species and different collectors, and (ii) the fact that the encysted parasites are distinctive in morphology, durably preserved alongside their hosts and can be detected and removed with minimal damage to archived hosts (including from fins). Our results indicated that, while *Scaphanocephalus* spp. has been present in Caribbean fish since at least 1948, the average infection load in surgeonfish and parrotfishes has increased by 49-fold from the historical period (1905–1966) to present (2017–2022). The ecological or evolutionary factors responsible for this increase–as well as its consequences for infected fish species remain conjectural. Available evidence indicates that *Scaphanocephalus* spp. infections are associated with reduced fish body condition, increased oxidative stress reactions, higher immunological activity and a decrease in market value [66,69], highlighting the potential ecological as well as economic repercussions of emergence. The ecological importance of surgeonfishes and parrotfishes in controlling macroalgae populations in coral reefs further underscores the need to evaluate these changes and their implications for reef community health.

## Data Availability

The data used as part of this study are archived on Figshare [81]. Supplementary material is available online [82].
